# Development and Experimental Study of Supercritical Flow Payload for Extravehicular Mounting on TZ-6

**DOI:** 10.3390/e26100847

**Published:** 2024-10-08

**Authors:** Liang Guo, Li Duan, Xuemei Zou, Yang Gao, Xiang Zhang, Yewang Su, Jia Wang, Di Wu, Qi Kang

**Affiliations:** 1 State Key Laboratory of Nonlinear Mechanics, Institute of Mechanics, Chinese Academy of Sciences, Beijing 100190, China; guoliang@imech.ac.cn (L.G.); yewangsu@imech.ac.cn (Y.S.); 2 School of Engineering Science, University of Chinese Academy of Sciences, Beijing 100049, China; duanli@imech.ac.cn; 3 Key Laboratory of Microgravity, Institute of Mechanics, Chinese Academy of Sciences, Beijing 100190, China; 4 Beijing Aerospace Command Control Centre, Beijing 100094, China; zxm1222@sina.com (X.Z.); gaoyang1862@163.com (Y.G.); 5 Beijing Institute of Spacecraft System Engineering, Beijing 100094, China; zhang_xiang97@126.com; 6 Beijing Key Laboratory of Engineered Construction and Mechanobiology, Institute of Mechanics, Chinese Academy of Sciences, Beijing 100190, China

**Keywords:** supercritical fluid, thermal capillary convection, microgravity environment, annular flow, out-of-cabin payload, temperature oscillation

## Abstract

This paper provides a detailed description of the development and experimental results of the supercritical flow experiment payload carried on the TZ-6 cargo spacecraft, as well as a systematic verification of the out-of-cabin deployment experiment. The technical and engineering indicators of the payload deployment experiment are analyzed, and the functional modules of the payload are shown. The paper provides a detailed description of the design, installation location, size, weight, temperature, illumination, pressure, radiation, control, command reception, telemetry data, downlink data, and experimental procedures for the out-of-cabin payload in the extreme conditions of space. The paper presents the annular liquid surface state and temperature oscillation signals obtained from the space experiment and conducts ground matching experiments to verify the results, providing scientific references for the design and condition setting of space experiments and comparisons for the experimental results to obtain the flow field structure under supercritical conditions. The paper provides a specific summary and discussion of the space fluid science experiment project, providing useful references for future long-term in-orbit scientific research using cargo spacecraft.

## 1. Introduction

In the microgravity environment, buoyancy convection is greatly weakened or disappears, while some secondary convection processes that are obscured on the ground, such as thermocapillary convection driven by surface tension, become prominent; in the microgravity environment, new mechanical systems can be studied, as well as new fluid physics mechanisms, including some “secondary effects” on the ground. The tilt method is a very important method for growing high-quality crystals, and the annular flow is a simplified model of the tilt method [1]. In a microgravity environment with a significant reduction in gravity, buoyancy convection is greatly weakened. When the free surface has a non-uniform distribution of temperature or concentration, the free surface will form a thermocapillary convection driven by surface tension, which directly affects the quality of material growth in space. Therefore, the study of thermocapillary convection driven by surface tension in a microgravity environment has very important practical applications [2].

The thermocapillary convection in the annular flow has very strong nonlinear characteristics, and in the theoretical analysis, the conditions selected are often too idealized, so the critical parameters obtained may not correspond very accurately to the experimental results. The study of the stability of the internal flow in a strongly nonlinear system is mainly dependent on experiments and numerical calculations to achieve it.

The initial research on the annular flow was concentrated on critical instability, and there was very little research on the flow phenomena and evolution process under large temperature differences. Garnier [3] studied the flow stability problem of thermocapillary convection in annular flows, using linear stability analysis to investigate hydrothermal waves, and confirmed that flow instability first occurred at the cold end. A three-dimensional numerical simulation of Prandtl number silicone oil was conducted to discover the existence of a flow transition process in thermocapillary convection [4]. Li [5], based on the method of asymptotic analysis, studied thermocapillary convection in a dual-layer coaxial annular flow model under microgravity, confirming that by adjusting parameters, instability in the model can be suppressed and can obtain analytical expressions for the flow field, temperature field, and pressure field in the model. Sim [6,7] numerically simulated the thermocapillary convection and transition process of the fluid in an inner-cooled annular liquid chamber, obtaining two different unstable flow patterns under the condition of a flat free surface and considering free-surface heat dissipation, and found that the temperature field structure could be expressed as a hydrothermal wave propagating circumferentially or radially. Shi [8] calculated the conditions for the generation of fluid waves in microgravity annular flows, analyzed the characteristics of thermocapillary convection and hydrothermal waves in the model with inner cooling, and found that when the Marangoni number was small, unidirectional propagating hydrothermal waves would spread over the entire region with time; when the Marangoni number was large, hydrothermal waves would propagate in different azimuthal directions.

With increasing space activities, significant progress has been made in in-orbit experiments on thermocapillary convection stability. Since 1992, Kamotani and Ostrach et al. [9] conducted the microgravity experiment on annular flow for the first time and observed the axisymmetric flow in the steady state. The experimental fluid was 10cSt silicone oil, the diameter of the liquid pool was 10 cm, and the depth was 5 cm. Two sets of heating systems were used; one was the laser heating system with a laser diameter of 0.5–3.0 cm, and the other was the electric film heating system with a film diameter of 1.11 cm [10]. Through the flow visualization technology (PTV), they observed the flow fields with different heating methods and free surface shapes and obtained 18 sets of experimental results, which were in good agreement with the corresponding numerical results. The obtained *Ma* = 3.1 × 10^5^ was five times that obtained in the ground experiment, but the oscillation had not been observed yet [11].

Kamotani and S. Ostrach [12,13] completed the second microgravity experiment in the USML-2 space laboratory. The experimental fluid was 2cSt silicone oil, and the diameters of the liquid pool were 1.2 cm, 2.0 cm, and 3.0 cm, respectively, with *Ma* < 6 × 10^5^ and the wall temperature of 14 °C. When the *Ma* number was high enough, the driving force of thermocapillary convection was mainly concentrated in the hot and cold zones, and the oscillation phenomenon was closely related to the flow in the hot zone [14]. The flow fields and temperature fields of the steady and oscillatory flows were observed, and the critical oscillation conditions of this space experiment were determined. The flow field was qualitatively observed, and the experimental result of thermocapillary convection in the 1.2 cm diameter liquid pool was consistent with the result of the ground experiment under normal conditions. This indicated that in experiments with equipment equal to or smaller than this size, the influence of gravity could be neglected. In the experiment, the surface deformation during oscillation was also observed, and a surface deformation parameter was defined. This parameter could well indicate the occurrence of oscillation and was independent of the heating rate. The isotherms obtained by the thermal camera discovered the changes in the surface temperature field during the oscillation; the influence of the critical temperature difference on the critical conditions was also studied, and the results showed that when the free surface was very curved, or the liquid layer was very shallow, the oscillation would be delayed. The influence of the heating rate on the temperature difference was further increased, and the pulsating mode of the two-earlobe oscillation pattern would occur in different areas of the free surface; the two-earlobe oscillation pattern was found in all experiments, and as more and more experimental and numerical simulations were conducted on the oscillatory flow in the annular pool, the three-earlobe pattern also appeared [15,16].

In 2006, Schwabe and Benz et al. reported the successful results of a set of experiments on thermocapillary convection in the annular region on the Russian FOTON-12 satellite [17,18]. The outer wall was heated with a radius of 40 mm, the inner wall was cooled with a radius of 20 mm, and the depth of the annular region was 2.5–20 mm. It showed that under a small horizontal temperature difference, the flow was a stable multi-cellular flow; as the temperature difference increased, the flow would lose its stability and first transform into hydrothermal waves, and then, as the temperature difference further increased, more complex oscillatory flow would occur. It also showed that as the thickness of the liquid layer increased, the number of flow cells in the multi-cellular structure decreased. The later numerical simulation results were consistent with the experimental results [19,20].

In recent years, the Key Laboratory of Microgravity Chinese Academy of Sciences has conducted extensive research on the annular flow problem, found the critical conditions for instability under a horizontal radial temperature gradient, and in-depth analyzed the flow patterns during the conversion process of buoyancy-thermocapillary convection [21,22]. In 2016, Kang et al. conducted a thermocapillary convection space experiment on the SJ-10 return satellite to study the convection instability problem under critical conditions. Due to the limitation of space resources, only 23 sets of experimental data were obtained, and in order to ensure the success of the space experiment and obtain effective scientific data, the fluid interface was maintained, and the maximum temperature difference was set lower. Only one experiment had a maximum temperature difference of 40 °C, and the maximum temperature difference in the remaining groups was no more than 35 °C [23].

Based on the in-depth study of thermocapillary convection, the authors found that the bifurcation routes leading to chaos in this flow system are very rich [24]. Kang et al. also discovered complex bifurcation coupling patterns in the ground-based circular flow comparative experiment [25]. In addition, Kang et al. discovered the new law of the comprehensive effect of the liquid bridge height-diameter ratio-volume ratio and the “jump” effect through the space experiment of the TG-2 liquid bridge thermocapillary convection on the Tiangong-2 space station. They found that the low-frequency mode starting region has multiple transitions, provided a complete conversion map of the liquid bridge traveling wave and standing wave, and explored the complex characteristics of the bifurcation road leading to chaos in the thermocapillary flow system [26]. Therefore, the space annular flow experiment should increase the temperature gradient to explore the bifurcation process at a higher Marangoni number, study the routes to chaos, and establish a strong correlation model between the chaotic roads and the flow mode [27,28]. The in-orbit experiment aims to study the space thermocapillary convection dominated by surface tension-driven flow, conducting multiple transitions, wave patterns, and multi-level evolution of space annular thermocapillary convection instability, as well as coupled bifurcation with large Marangoni number. It is a key project supported by the National Natural Science Foundation of China. The cargo spacecraft is an excellent microgravity experiment platform, enabling the exploration of universal laws of space thermocapillary convection evolution under microgravity conditions, which is of great significance for the formation, development, evolution, and chaos of fluid flow, as well as the formation mechanism of turbulence. By taking advantage of the opportunity to carry out the Key Program of the National Natural Science Foundation, we can achieve the optimal allocation of resources for both parties and achieve a win–win situation for space technology and scientific research. It also provides important reference values for subsequent scientific experiments conducted outside the spacecraft cabin under extreme conditions.

We conducted a total of 47 space experiments, and it was also the first time we conducted space science experiments outside the cargo spacecraft. We obtained the space domain pattern and temperature field oscillation under supercritical conditions, verifying the feasibility of conducting space science experiments outside the cabin and expanding the experimental platform for space science experiment research.

## 2. Research Methods and Experimental Plan

### 2.1. Experimental Model and Working Principle

The supercritical load studies problems such as flow transition under high Marangoni numbers, which is when the fluid is under ultra-high critical conditions. By observing phenomena such as flow pattern transformation, temperature oscillation, and surface oscillation under different volume ratios, the transition conditions and transition process of the flow are studied, and the interface tension-driven convection and flow boundary layer, the instability mechanism of the convection, the oscillation law and transition process of the convection, and the propagation law of fluid waves are explored.

The studied model is an annular liquid pool. By applying temperatures to the central column and the outer wall of the annular liquid flow model within the load (the temperature of the central column is *T_H_*, the temperature of the outer wall is *T_L_*, and the temperature difference is Δ*T* = *T_H_* − *T_L_*), a temperature difference can be established, thereby driving the convection of the fluid inside the liquid pool. By increasing the temperature difference, the evolution law of the fluid flow can be observed, from the steady flow becoming unstable to the periodic flow and finally developing into chaos or even turbulence. The liquid is controlled by the surface tension within the channels between the inner and outer diameters. The volume ratio effect can be studied by changing the volume of the liquid between the central column and the outer wall.

### 2.2. Load Development

The load operates autonomously and independently and is controlled by the Beijing control center to implement in-orbit experiment instructions and conduct scientific experiments in the field of space fluid physics; data is transmitted to the ground via the cargo spacecraft and space station link, and the Beijing control center, the fifth aerospace institute, and the space application center work together to complete the in-orbit experiment status telemetry, scientific experiment data downlink reception and distribution, and analysis by the scientific experiment team to obtain related scientific results.

#### 2.2.1. Box Structure Design

**Figure 1:**
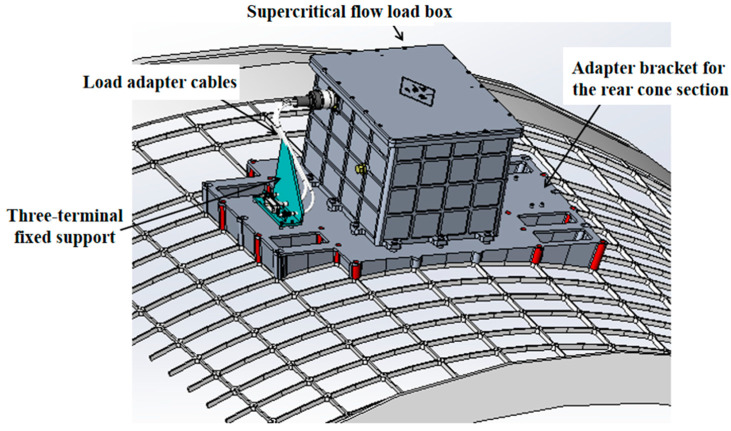
Load assembly (load and adapter bracket, without thermal wrap).

The supercritical flow load carrier is located at the rear conical section in the starboard quarter of the TZ-6 cargo spacecraft’s hull, with a volume of 370 × 300 × 235 mm^3^. As shown in Figure 1, it is connected to the hull through a conical section adapter bracket.

**Figure 2:**
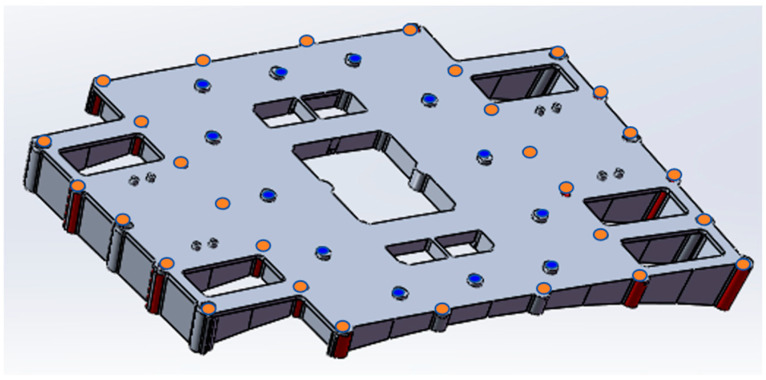
Adapter bracket (excluding thermal wrap).

Since the ship’s outer surface is curved, the load box cannot be fixed directly. A transition bracket is needed to assist in fixing the load box. The bracket design is shown in Figure 2, based on the hole positions and wiring locations on the hull. The inner ring has 12 blue hole positions that are fixed to the load box with bolts, and the outer ring has 19 orange hole positions that are connected to the hull. The lower part of the bracket is curved to fit the hull. The combined assembly’s outer contour dimensions are 720 × 450 × 320 mm^3^, meeting the maximum contour requirements.

**Figure 3:**
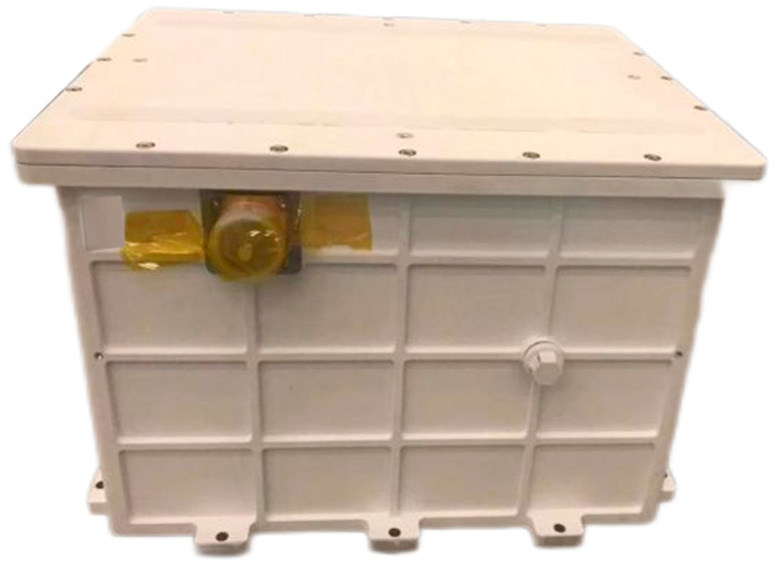
Payload Body.

The load box has one electrical interface, which has been designed with strong and weak electrical isolation to provide power and communication functions. In addition, a three-terminal fixing bracket has been installed on the transition bracket to separate the 1553B and Ethernet communication zones. The load box is equipped with a pressure relief valve to complete the vacuum and pressure-holding actions, as shown in Figure 3.

Because it is exposed to outer space and directly receives radiation from the sun, in order to ensure that the components within the payload operate normally, a thermal enclosure needs to be designed on the outside of the payload. This payload uses a design with symmetric heat dissipation from the two side faces, with thermal enclosures on the top, bottom, and the remaining two sides. The top is covered separately, while the other three sides are covered with thermal enclosures. This can maintain the internal payload at a suitable temperature range, ensuring the stability of the single experiment condition.

The main performance technical indicators are as follows:

Power supply interface: 100VDC power supply, the payload is transformed to 28 V.

(1) Payload operating power consumption: average power consumption not exceeding 50 W, peak power consumption not exceeding 85 W;

(2) Payload thermal insulation power consumption: average power consumption not exceeding 180 W, peak power consumption not exceeding 240 W.

Weight: 28.5 kg

Volume: 370 × 300 × 235 mm^3^ (payload box body); 385 × 315 × 250 mm^3^ (payload box including thermal enclosure)

Liquid pool: inner radius 4 mm, outer radius 20 mm, height 12 mm;

Volume ratio: 0.6–1.05;

Maximum temperature difference: 60 °C+.

#### 2.2.2. Payload Function and Performance Introduction

**Figure 4:**
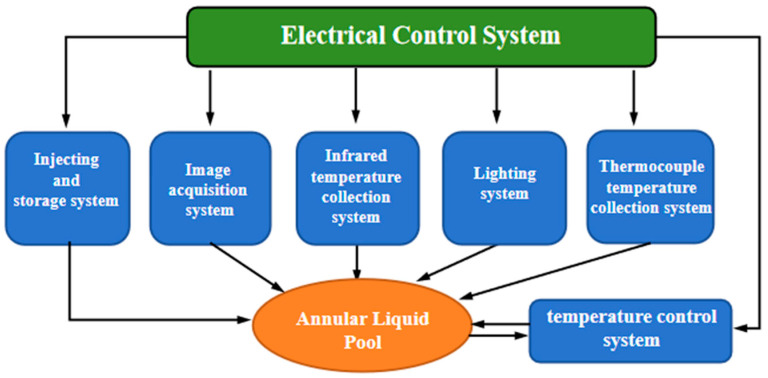
Composition of the supercritical flow load.

The supercritical flow load includes several technical units, as shown in Figure 4, which will perform functions such as fluid injection, temperature control, electrical control, high-speed data retransmission, image acquisition, temperature measurement, and illumination.

**Figure 5:**
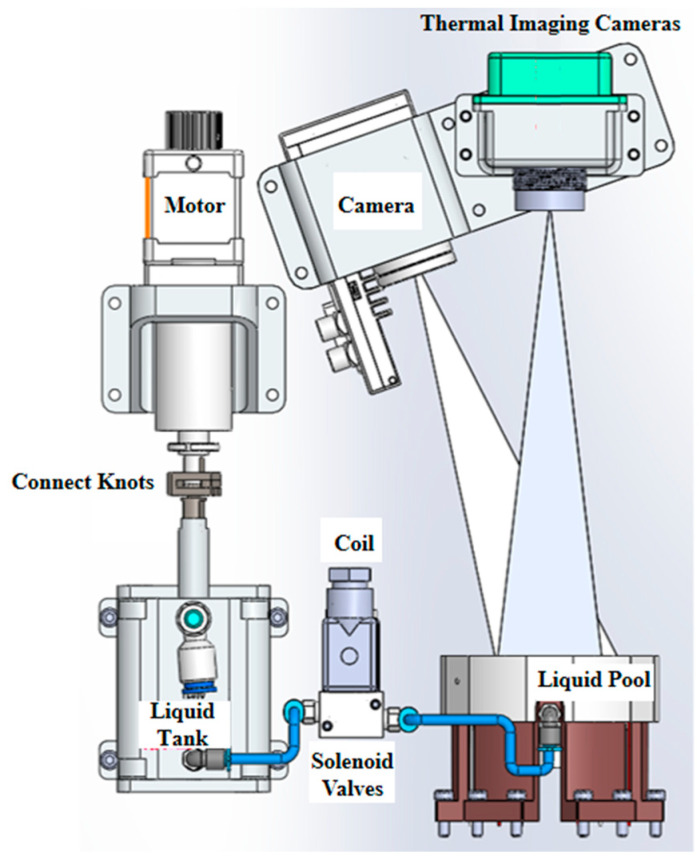
Schematic diagram of the supercritical flow model.

The load established the annular flow experimental fluid systems with different volume ratios by controlling the liquid injection and using a camera to observe the fluid interface of the model, as shown in Figure 5. Through the active temperature control of the central column and the outer wall of the liquid pool, the transition and bifurcation of the thermocapillary convection of the fluid system and the development of turbulence under large Marangoni numbers were achieved. The evolution of the internal temperature of the fluid was measured by using high-precision thermocouples, and the distribution of the temperature field of the free interface of the fluid was obtained by using an infrared camera so that the scientific research on the bifurcation routes and mode conversion of the thermocapillary convection flow under different volume ratios can be completed.

**Figure 6:**
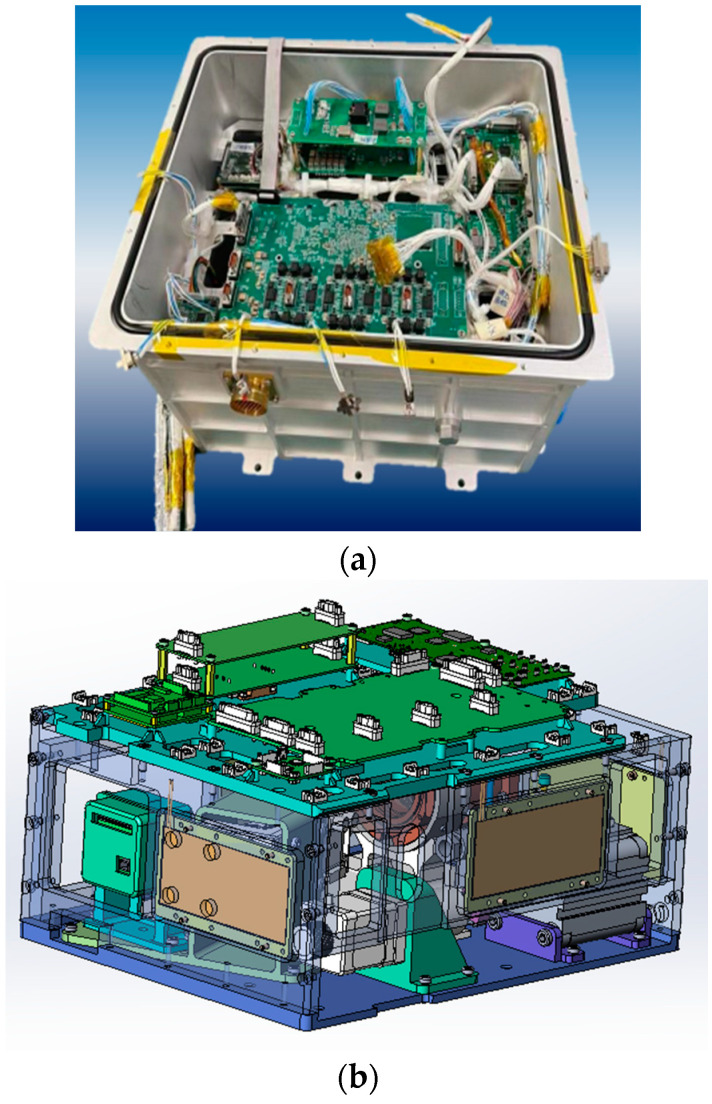
Three-dimensional structure diagram. (**a**) Structure diagram of the load. (**b**) Internal layout.

The load is divided into two layers, as shown in Figure 6, with the fluid model and observation system in the lower layer and circuit boards in the upper layer. The bearing design between the partition and the box allows the partition to be opened to a certain angle for confirmation of the load state.

**Figure 7:**
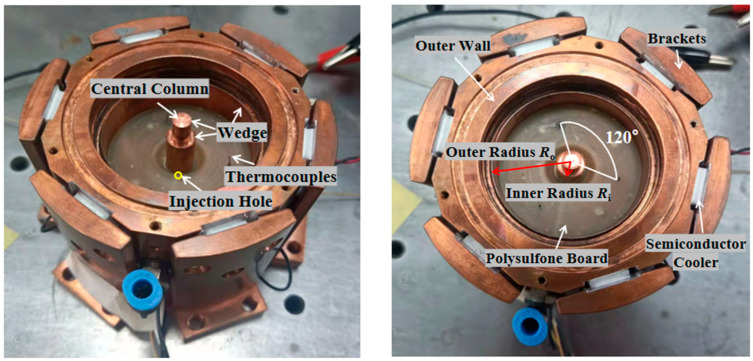
Physical model of the fluid.

The experimental unit device is composed of the following functional modules:

Liquid pool module—the fluid physics model of the load: annular liquid pool, as shown in Figure 7. Referring to the annular flow model of SJ-10, a flow channel width of *R_o_* − *R_i_* = 16 mm is adopted. The radius of the central column (also the heating column) is *R_i_* = 4 mm, the inner radius of the outer wall of the liquid pool is *R_o_* = 20 mm, and the depth of the liquid pool is *d* = 12 mm. Both the central column and the outer wall of the liquid pool are made of copper. An electric heating film heats the central column, and 6 semiconductors are attached to the outer wall of the liquid pool. There are 6 brackets outside the cooling plates for heat transfer and fixing with the box. The bottom of the liquid pool is a polysulfone board with good thermal insulation. A liquid injection hole is reserved at the bottom of the polysulfone board and is connected to the liquid cylinder through pipelines.

In the space environment, due to the absence of gravity, the maintenance of liquid surfaces is a key technical problem. There are many anti-crawling designs for liquid pool systems. The liquid pool’s central column and side walls have anti-crawling spikes and other structures, and a crawl-resistant liquid is applied to the top of the spikes.

KF96L 2cSt silicone oil (*Pr* = 28.57; thermal diffusion coefficient *κ* = 7.00 × 10^−8^ m^2^/s; surface tension coefficient *σ_T_* = −7.15 × 10^−5^ N/(m·K); density *ρ* = 873 kg/m^3^; kinematic viscosity *ν* = 2 × 10^−6^ m^2^/s) is chosen as the experimental working fluid.

The storage and injection modules consist of a liquid cylinder, an injection motor (PI motor), an electromagnetic valve, and pipelines, which are used to control the amount of liquid injected into the liquid pool by setting the position of the motor and achieving a specific volume ratio.

The internal cylindrical diameter of the liquid cylinder is φ = 40 mm, the effective stroke is 25 mm, and the effective volume is 31.4 mL, which is three times the volume of the liquid pool, ensuring a margin of safety. The liquid injection motor is a motor with the model M227 produced by the PI (Physik Instrumente) Company, Karlsruhe, Germany. Its stroke is 25 mm, and the minimum movement displacement is 0.05 μm.

Silicone oil is stored in the liquid cylinder before the experiment begins; during the experiment, the PI motor pushes the liquid cylinder to inject a liquid, and the controlled injection volume can achieve different volume ratios *V*/*V*_o_ = 0.6, 0.7, 0.8, 0.9, 1.0….

**Figure 8:**
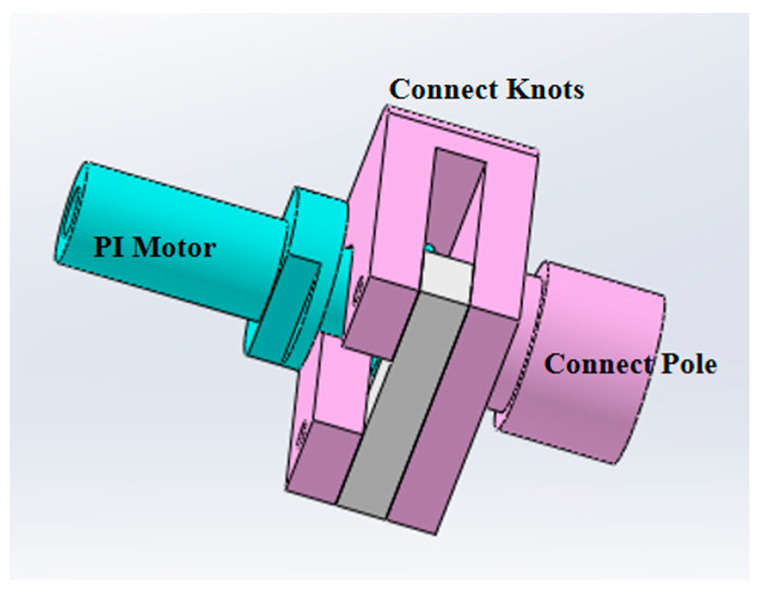
Connection joint design diagram.

To prevent the motor from moving beyond its limit or overshooting during launch, causing the motor to jam, etc., the load is specifically designed with a connection joint that can achieve bidirectional adjustment of the motor’s movement (as shown in Figure 8).

The dual-channel temperature control module uses the PID algorithm to control the temperature of the center column and outer wall of the load separately, and the high and low temperature, heating time, etc., can be set separately. Three K-type thermocouples are installed on the central column and the outer wall of the liquid pool, respectively. The median value of the three thermocouples is taken as the temperature of the central column and the outer wall and fed back to the temperature control system to achieve control over the temperature, heating time, etc. The bare wire diameter of the thermocouple is 0.127 mm, and the accuracy is ±0.5%*T*_t_, where *T*_t_ is the temperature measured instantly.

Infrared temperature acquisition module—the infrared camera can complete infrared temperature field measurement in real-time. This payload can achieve high-speed Ethernet transmission by using an IP address. The infrared camera adopts the TAU2 uncooled infrared camera of FLIR company, with a resolution of 336 × 256 pixels, a sensitivity of no more than 50 mK, and a sampling frequency of 7.5 Hz.

Thermocouple temperature measurement module—At the position of *r* = (*R_o_* + *R_i_*)/2 within the liquid pool, there are three K-type thermocouples. The bare wire diameter of the thermocouples is 0.08 mm, and the accuracy is also ±0.5% *T*_t_ (where *T*_t_ is the temperature measured instantaneously). The three thermocouples are spaced 120° apart from each other, with a sampling rate of 20 Hz, and they are capable of capturing the details of fluid fluctuations.

Image acquisition and lighting module—There are two LED lights in the payload. One is located at the underside of the camera and is used for supplementing light for the camera. The other one is at the bottom of the liquid pool and is used for illuminating the liquid pool. The camera adopts a CMOS sensor of model WAT −30 H from WATTEC company, Vlaanderen, Belgium. The effective resolution is 1920 × 1080 pixels, and the sampling frequency is 30 Hz. It is used to record the liquid injection process and flow conditions of the liquid pool.

Electronics control module—It controls individual components and completes the operation of the process.

Box heating and temperature control module—This module is designed to adjust the internal temperature of the box. A heating film is attached to each of the six inner surfaces of the box, and the designed power is 60 watts. A thermocouple (the same as the thermocouples at the central column and the outer wall of the liquid pool) is attached to each inner wall surface of the box to measure the box temperature. At the start of the experiment, if the box temperature is less than 0 °C, the heating film operates to heat the box to 0 °C and maintain this temperature; when the box temperature is greater than 0 °C, the electric heating film on the box does not operate.

Please refer to Appendix A for the list of specific equipment in Table A1.

#### 2.2.3. Parameter Definition

The factors that determine the motion state of the flow field mainly include the geometric dimensions of the liquid layer, the radius of the heating column *R_i_*, the inner radius of the outer ring *R_o_*, and the depth of the liquid pool *d*; the physical property parameters of the fluid such as the thermal diffusivity coefficient *α*, the volume expansion coefficient *β*, the dynamic viscosity coefficient *μ*, the density *ρ*, the surface tension temperature coefficient *σ_T_*, and the internal and external temperature difference Δ*T* of the liquid pool. These influencing factors are combined into dimensionless parameters with different physical meanings to control the flow state.

The Marangoni number characterizes the relative magnitude relationship between the thermocapillary convection effect and the viscous and thermal diffusion effects. When the Marangoni number is relatively large, the convection effect caused by the surface tension gradient is dominant, while when the Marangoni number is relatively small, the viscous and thermal diffusion effects are relatively more important.

(1) $$Ma=\frac{\sigma_{T}\left(T_{H}-T_{L}\right)\left(R_{o}-R_{i}\right)}{\mu \kappa },\;\mathrm{describes}\;\mathrm{the}\;\mathrm{driving}\;\mathrm{effect}\;\mathrm{of}\;\mathrm{surface}\;\mathrm{tension}.$$

*σ_T_* is the rate of change of surface tension with temperature, representing the sensitivity of surface tension change caused by temperature change;
*T_H_* − *T_L_* is the characteristic temperature difference, reflecting the degree of temperature non-uniformity in the model;
*R_o_* − *R_i_* is the characteristic length;
*μ* is the dynamic viscosity of silicone oil, reflecting the ability of silicone oil to resist deformation;
*κ* is the thermal diffusivity coefficient of silicone oil, describing the speed of heat diffusion in the fluid.

The temperature dependency of the kinematic viscosity of the working fluid is evaluated from the following equation:

(2) $$\frac{\nu }{\nu_{25}}=\exp\left(5.892\frac{25-T}{273.15+T}\right),$$

(3) $$\nu =\frac{\nu \left(T_{H}\right)+\nu \left(T_{L}\right)}{2},$$

where *v*_25_ and *T* is the kinematic viscosity at 25 °C and the temperature considered, respectively.

Under the action of surface tension, the liquid interface presents a curved interface. Spatial experiments use the volume ratio to describe the interface morphology.

*Vr* = *V*/*V*_0_, where *V* is the liquid volume and *V*_0_ is the volume between the channels of the liquid pool.

Among them, *V*_0_ = π(*R_o_*^2^ − *R_i_*^2^)*d.*

#### 2.2.4. Spacecraft Extreme Condition Adaptability Design

Structure design

**Figure 9:**
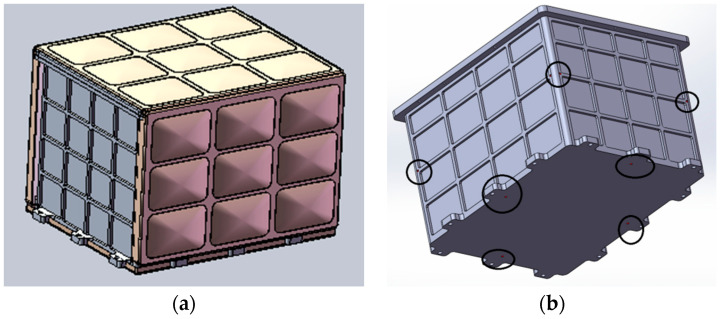
Thermal wrapping structure design. (**a**) Thermal cladding outline drawing; (**b**) thermal cladding mounting hole.

In order to ensure that the payload’s environmental temperature fluctuation is not too large, the normal operation of the components is ensured, and a relatively stable environment was maintained in one experiment, and the payload was designed with thermal wrapping. As shown in Figure 9, the specific details are as follows:

(1) The payload uses 2A12 material, with internal cathode conductive white, emissivity 0.87, and solar absorptivity 0.13;

(2) The base plate of the box is insulated and installed between the box and the spacecraft;

(3) The outer surface of the cabinet wall panels is covered with multiple layers of thermal insulation components and anti-atomic oxygen flame-resistant fabric.

The multiple layers of thermal insulation components and anti-atomic oxygen fabric are sewn together as a whole, with a maximum thickness of 10 mm.

The wrapping material is designed with multiple layers:

(1) The intermediate layer uses 15 layers of 20 μm thick double-sided aluminum-coated polyimide film materials with an aluminum coating thickness of no less than 40 nm;

(2) The outer film uses a single-sided aluminum-coated polyimide film, with the insulation layer facing out;

(3) The inner film uses a double-sided aluminum-coated polyimide film;

(4) The multi-layer material should have ventilation holes, with the area of the holes accounting for 0.5%~1% of the total area and the hole diameter of 1~3 mm;

(5) Each unit of the multi-layer thermal insulation component needs to be riveted with a conductive film, and the free end of the conductive film is welded to Φ = 4.2 mm military soldering clip;

(6) The outer cover: The multi-layer thermal insulation material is wrapped around the outer surface of the four wall panels of the experiment chamber, and a white atomic oxygen-resistant flame-retardant fabric of the SFGF-W(K) type is used to cover the area outside the wrapping material.

The wrapping material is sewn together as a whole and wrapped around the four wall panels of the experiment chamber in a manner of overall wrapping. Four hook-surface nylon fasteners are fixed on each experiment module panel at least, which are fixed by using GD4-14 C silicone adhesive. The corresponding hook-surface nylon fasteners are sewn on the inner side of the multi-layer material at the corresponding position. The experiment module panel is provided with 12 M4 threaded holes near the edge for connecting the ground wires of the multi-layer thermal insulation film.

**Figure 10:**
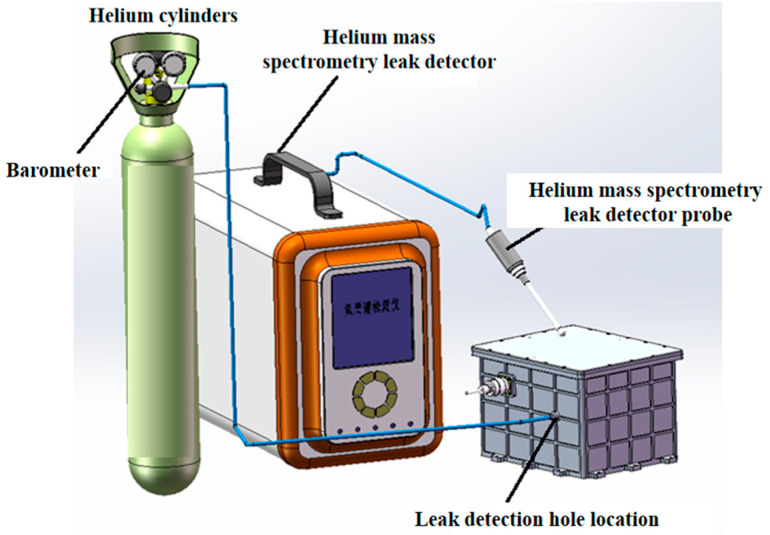
Leak detection system.

Figure 10 shows the Helium Leak Test. A pressure regulator limits the helium gas in the high-pressure gas cylinder and then passes through a high-pressure pipe to the quick-release pipe valve on the box body, which is connected to the box body. The box body is pressurized, and the pressure value can be read from the pressure gauge on the helium gas cylinder. At the same time, the helium mass spectrometer leak detector is opened to detect whether there is a leak. The position of the leak detection hole on the box body is shown in the figure, which is an M8 threaded hole. The corresponding quick-release pipe valve thread specification is also M8 and the corresponding plug thread is also M8. The sealing plug seals the box body by squeezing the O-ring between the thread and the plug to achieve the sealing effect. The designed leakage rate is ≤3.5 × 10^−6^ Pa·m^3^/s, and the test results show that the leakage rate is ≤3.3 × 10^−6^ Pa·m^3^/s.

Software design

The supercritical flow payload needs to complete the actions of electromagnetic valve switching, motor movement, temperature control, video data acquisition, data packaging and storage, and downlink according to the predetermined program.

There are two types of in-orbit space experiments: real-time transmission and storage and downlink.

The fixed experimental flow is generally shown in Table 1.

The experiment start X in the table, X is a variable representing different experiment conditions, including high temperature, low temperature, temperature rise time setting, motor movement parameters, infrared camera collection parameters, and thermocouple collection parameters.

Due to the time resource limitation in space experiments, the most suitable experiment method will be selected according to the actual in-orbit situation.

## 3. Ground Verification Experiment

In order to verify whether the payload has the functions and performance required for conducting space on-orbit experiments, payload ground experiments have been carried out on the ground. In order to achieve the flow under supercritical conditions in the ground experiment, the flow of a thin liquid layer with a liquid layer thickness ≤5 mm was observed, that is, the thermocapillary convection with a volume ratio ≤0.42.

**Figure 11:**
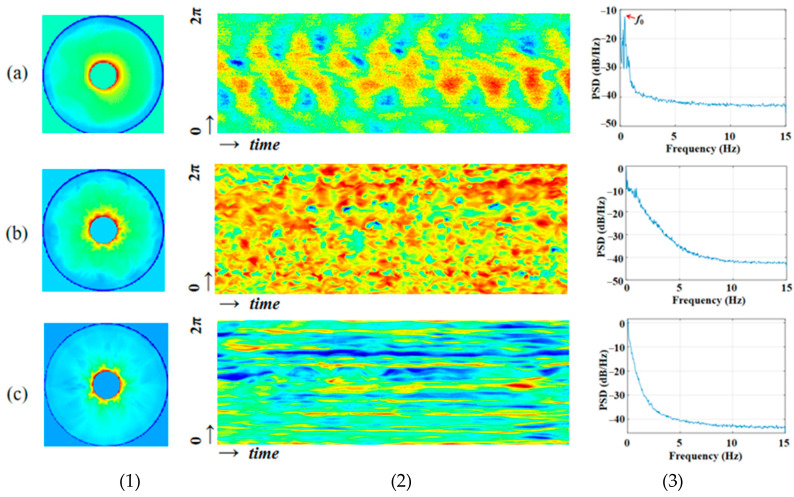
Ground verification experiment. (**a**) Weakly supercritical periodic oscillation −Δ*T* = 20 °C, *Vr* = 0.25, *Ma* = 1.87 × 10^5^ (surface tension dominated); (**b**) weakly supercritical periodic oscillation −Δ*T* = 50 °C, *Vr* = 0.25, *Ma* = 4.68 × 10^5^ (surface tension dominated); (**c**) non-oscillatory flocculent structure due to buoyancy −Δ*T* = 50 °C, *Vr* = 0.42, *Ma* = 4.68 × 10^5^ (buoyancy flow dominated). (1) Infrared images; (2) temperature-time evolution in the radial direction; (3) spectrum diagram.

A linear heating method was used in the ground verification experiment. As shown in Figure 11, the flow field fluctuation conditions under different temperature differences and different volume ratios can be obtained. In a thin liquid layer with a volume ratio of 0.25 and a temperature difference of 20 °C, the surface tension plays a dominant role, and the flow is in a state of periodic oscillation under weak critical conditions. The temperature gradient is obvious in the radial direction from the central column to the outer wall, and there is only one main frequency with extremely high energy. It can be known from the circumferential time evolution that it is a hydrothermal wave with circumferential propagation characteristics. The bright and dark stripes represent the peaks and troughs of the temperature. Each wave has a specific wave number. Two waves with the same frequency and amplitude and propagating in opposite directions form a standing wave.

In a liquid pool with a volume ratio of 0.25 and a temperature difference of 50 °C, the flow has entered a periodic oscillation under weak supercritical conditions. Currently, multiple frequencies with relatively high energy are in the flow field. There is a gradient in the circumferential temperature, and the flow field structure is shown in Figure 11b, which is in an extremely disordered state. The hydrothermal wave has a more complex structure, with more detailed petal-like structures at the original peaks and troughs. In addition to the circumferential and axial directions, the flow is more random.

When maintaining a temperature difference of 50 °C as unchanged, in a thick liquid layer with a volume ratio of 0.42, the buoyancy flow dominates the flow, and the periodicity is lost. The temperature distribution of the temperature field is radial, similar to flocs emitted from the central column. From the time evolution, it can be seen that the high temperature and low temperature show an uneven distribution state, and only small changes occur over time, and the influence of environmental disturbances cannot be excluded.

**Figure 12:**
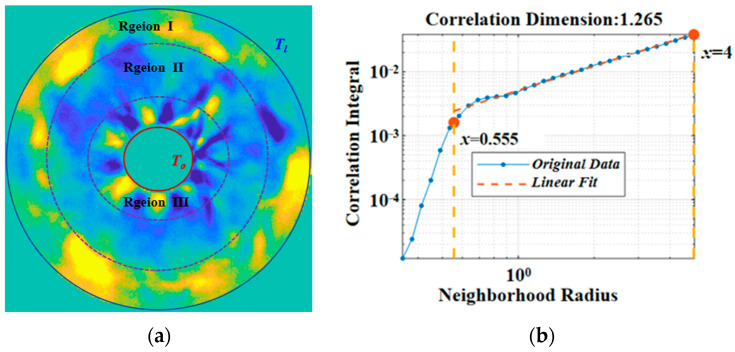
Correlation dimension at large temperature difference. (**a**) Infrared image (*Ma* = 5.62 × 10^5^); (**b**) correlation dimension.

As shown in Figure 12, thermocapillary convection under supercritical conditions was observed in the ground experiment. There are abundant flow phenomena when the volume ratio is 0.25 and the temperature difference is greater than 60 °C. By introducing the chaotic dynamics theory, the correlation dimension analysis under the supercritical state can be completed based on the phase space reconstruction. The interface temperature gradient becomes larger, the flow is more intense, the temperature field has a more complex structure, and different from the weak critical condition, a “reverse temperature difference distribution state” of temperature appears. Region I is a hot region, Region II is a cold region, and Region III is a region of alternating hot and cold. That is to say, the heat exchange at the near-hot end of the flow is more regular, and a high-temperature region appears at the cold end. Due to heat conservation, a low-temperature region appears in Region II, and the flow distribution is more complex. The flow field seems to be chaotic, but in fact, it is regular. The obtained correlation dimension is 1.265; that is, the flow has entered a chaotic state (Figure 12b). These phenomena indicate that the load can establish a fluid physical model and successfully carry out the experimental process to obtain valid scientific data.

## 4. Space Experiment Research and Result Analysis

After analyzing the experiment model, payload state, and downlink link, the project team determined four experiment modes and four sets of experiment conditions (the original plan was two sets of experiments + two sets of expanded experiments). Starting from December 2023, in order to obtain effective experimental data, the experiment will be conducted in a “do experiment + downlink + store” manner, and the experiment flow and signal acquisition time of CCD and other devices will be adjusted to match the data generated. The payload’s in-orbit experiment operation was normal, and 47 effective in-orbit experiments were conducted. There are four experimental modes:

Mode 1: Time-segmented + Camera + internal temperature measurement + surface temperature measurement + cooling process-1, abbreviated as Camera + internal + surface + cooling-1: heating rate of 1 °C/min, maximum temperature difference of 50 °C, as shown in Table 2 and Table 3.

Mode 2: Camera + internal +surface + cooling-2: heating rate of 1 °C/min, maximum temperature difference of 50 °C, as shown in Table 2 and Table 3.

Mode 3: Camera + internal +surface-3: heating rate of 0.6 °C/min, maximum temperature difference of 60 °C, as shown in Table 4 and Table 5.

Mode 4: Camera + internal +surface-4: heating rate of 0.6 °C/min, maximum temperature difference of 60 °C, as shown in Table 4 and Table 5.

**Figure 13:**
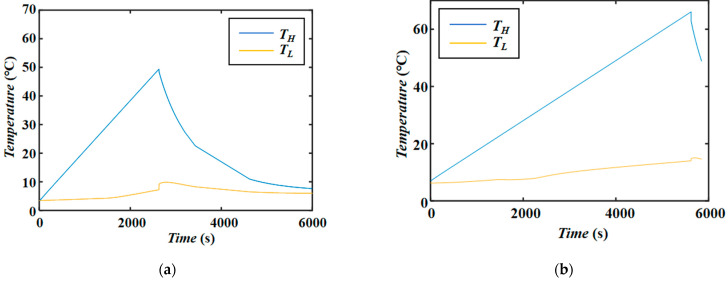
Temperature control curves under different heating rates. (**a**) 1 °C/min heating curve (corresponding to modes 1 and 2); (**b**) 0.6 °C/min heating curve (corresponding to modes 3 and 4).

**Figure 14:**
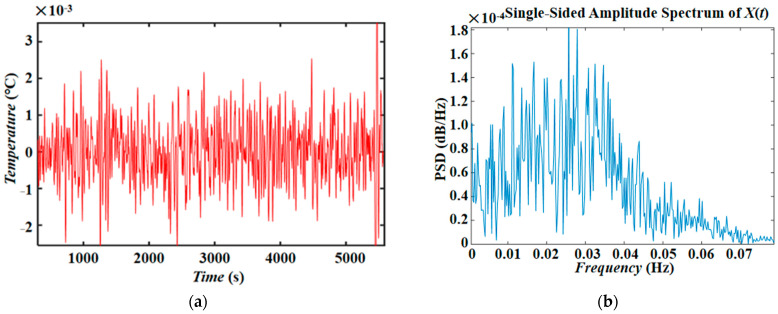
Thermocouple temperature signal and spectral analysis. (**a**) 1 °C/min heating curve (corresponding to modes 1 and 2); (**b**) 0.6 °C/min heating curve (corresponding to modes 3 and 4).

Experiments will be conducted daily 1–3 times, with each experiment lasting about 4–6 h, with a 2 h interval between experiments to allow the payload to recover to its initial state. During the experiment, the experiment status can be judged through telemetry parameters, and the scientific results can be preliminarily analyzed. Each experiment will generate 3–6 GB of supercritical flow space experiment data. Through the space experiment data, the transition process of supercritical flow in different experimental modes can be analyzed:

(1) High-precision thermocouples were used to measure temperature changes under different heating modes. The rapid heating rates (1 °C/min) of modes 1 and 2 are shown in Figure 13a, and the slow heating rates (0.6 °C/min) of modes 3 and 4 are shown in Figure 13b. The hot end temperature control has excellent linearity, providing a uniform temperature gradient for the supercritical flow load. Figure 14 shows the internal temperature oscillation signals and spectral analysis of the thermocapillary convection under supercritical conditions in the experiment, which has important scientific value for studying the supercritical chaotic transition process.

(2) The surface temperature distribution of the experimental fluid model was obtained using a thermal imager, and the process of establishing temperature gradients was clearly observed. We have obtained high-quality surface temperature images, clearly recording the radial wave distribution pattern and the evolution process of the scientific data.

The temperature difference increases, periodic fluctuations begin to appear in the flow, oscillation phenomena occur in the flow field, and hydrothermal waves with *m* = 3 appear in the temperature field. The cold and hot regions are alternately and evenly distributed in the temperature field (Figure 14b). When the temperature difference increases again, the periodicity of the flow disappears, and the hydrothermal waves in the temperature field present a state of “fragmented” distribution of cold and hot regions. Different from the ground experiment (Figure 12), due to the greatly weakened gravitational effect, the temperature distribution of thermocapillary convection under microgravity is more complex.

**Figure 15:**
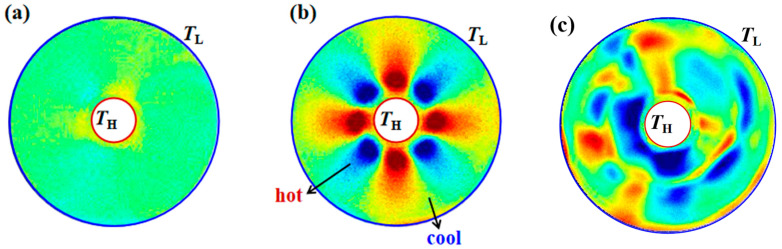
Evolution of the surface temperature field measured by an infrared camera, *Vr* = 0.85. (**a**) Steady flow (Δ*T* = 5 °C, *Ma* = 4.68 × 10^4^); (**b**) periodic oscillation (Δ*T* = 25 °C, *Ma* =2.34 × 10^5^); (**c**) supercritical flow (Δ*T* = 60 °C, *Ma* =5.62 × 10^5^).

**Figure 16:**
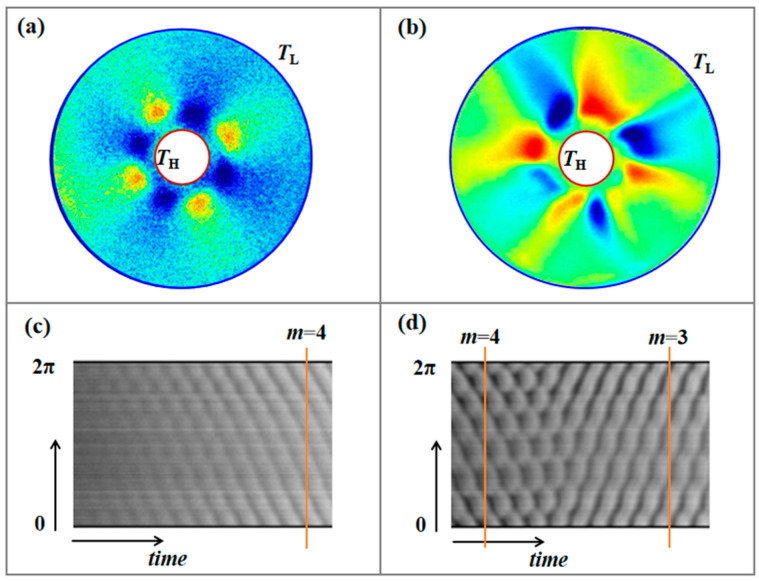
Evolution of annular flow surface waves, *Vr* = 0.95. (**a**) Hydrothermal waves with wave number *m* = 4; (**b**) coupled hydrothermal waves with wave number *m* = 3 and *m* = 4; (**c**) circumferential temperature—time evolution of the hydrothermal waves with *m* = 4 in (**a**); (**d**) circumferential temperature—time evolution of the coupled waves in (**b**).

Long-term observation of the temperature perturbation field during the heating process can be carried out through the data of the infrared camera to analyze the spatio-temporal evolution law of the transition from the thermocapillary convection oscillation mode to chaos under the condition of large Marangoni number in the microgravity environment. The critical conditions consistent with infrared observations are measured by using the thermocouple inside the annular flow. Space experiments show that when the temperature difference exceeds the critical value, the convection evolves from axisymmetric flow instability to circumferential hydrothermal waves. The time evolution analysis can more intuitively display the transition process of the annular flow and obtain the physical images of microgravity interface flow instability under the influence of different volume ratios and geometric parameters. As shown in Figure 15 and Figure 16, there is a competition of instability modes with wave numbers *m* = 4 and *m* = 3 in the temperature field. The oscillation of the thermocapillary convection in the annular flow is mainly circumferential fluctuations with *m* = 4. With the increase of the temperature difference, a complex surface fluctuation structure coupled with *m* = 3 and *m* = 4 appears. Space experiments reveal that the coupling effect of different wave numbers is the hydrodynamic essence of the transition from thermocapillary flow to chaos, which is of great scientific research value for understanding space interface instability and turbulence formation and is of great significance for guiding the application of flow control in space crystal growth.

## 5. Summary

This paper introduces the development and experimental situation of supercritical flow load. The design schemes of the load box, the adapter bracket, and the thermal wrap are introduced during the development of the cargo spacecraft’s external load, and the detailed process of conducting scientific experiments on the cargo spacecraft’s external load in orbit is explained using a specific operational scenario. The space experiment exceeded its expected goals and obtained a wealth of valuable space experiment data. Ground verification experiments were conducted to obtain the flow modes under supercritical and super-high-critical conditions, perform modal decomposition, and conduct flow field analysis. Based on the reconstruction of phase space, an attractor of chaos is introduced, the flow motion development trajectory is studied, the correlation dimension is calculated, and chaotic flow states are identified. Quantitative analysis of the flow is conducted to provide scientific reference and basis for the arrangement of in-orbit experiment conditions, the selection of silicone oil models, scientific observation means, and data processing and analysis methods. It also provides ground data for comparative analysis of the scientific achievements of space experiments. The scientific data obtained from this in-orbit experiment is of great scientific significance for exploring the basic physical laws of the formation, development, evolution, and chaos of space thermocapillary convection flow and the mechanisms of turbulence formation. It also has guiding value for the application research of the Czochralski crystal growth method. This project is the first to conduct microgravity fluid science experiments outside the cabin of a cargo spacecraft, exploring and expanding the microgravity experimental platform and space environment conditions for fluid physics research.

## Figures and Tables

**Table 1:** Fixed experimental process.

No.	Event	Time	Note
1	100 V 7th path connected (power on for supercritical flow payload)	T0	First experiment execution
2	Heating film for box body enabled	T0 + 1 min	
3	Heating film for box body disabled	T1	T1 − T0 > 31 min
4	Modify IP address 2	T1 + 20 s	
5	Modify gateway 2	T1 + 40 s	
6	Modify network port 1	T1 + 60 s	
7	Modify network speed 1	T1 + 80 s	
8	Select storage area 1	T1 + 100 s	
9	Set motor torque 1	T1 + 120 s	
10	Motor current position injection 1	T1 + 210 s	
11	Camera lighting brightness setting 1	T1 + 300 s	
12	Liquid pool lighting brightness setting 1	T1 + 320 s	
13	Experiment start X	T1 + 340 s	
14	Data readout enable T2	T2	T2 − T1 > 2.5 h
15	Load RT1 test data transmission allow	T2 + 20 s	
16	Ethernet data transmission starts		Every time entering the telemetry area
17	Ethernet data transmission stops		Every time leaving the telemetry area
18	Load RT1 test data transmission prohibited		Data transmission complete and stop

Note: The times in the table are preset times, and the specific times can be adjusted according to the flight control conditions.

**Table 2:** Group 1 experiments (9 experiments).

No.	New Experiment	Motor Position (mm)	Heating Rate (°C/min)	Maximum Temperature Difference (°C)	Experimental Mode	Duration (min)
1	1.5	6.912	1	50	Mode 1	90
2	1.6	8.064	1	50	Mode 1	90
3	1.7	9.2160	1	50	Mode 1	90
4	1.8	9.792	1	50	Mode 1	90
5	1.10	10.368	1	50	Mode 1	90
6	1.11	10.944	1	50	Mode 1	90
7	1.12	11.520	1	50	Mode 1	90
8	1.13	12.096	1	50	Mode 1	90
9	1.14	12.672	1	50	Mode 1	90

**Table 3:** Group 2 experiments (18 experiments).

No.	New Experiment	Motor Position (mm)	Heating Rate (°C/min)	Maximum Temperature Difference (°C)	Experimental Mode	Duration (min)
1	1.16	13.824	1	50	Mode 1	90
2	1.17	13.824	1	50	Mode 2	90
3	1.18	14.976	1	50	Mode 1	90
4	1.19	14.976	1	50	Mode 2	90
5	1.20	15.552	1	50	Mode 1	90
6	1.21	15.552	1	50	Mode 2	90
7	1.22	16.128	1	50	Mode 1	90
8	1.23	16.128	1	50	Mode 2	90
9	1.24	16.704	1	50	Mode 1	90
10	1.25	16.704	1	50	Mode 2	90
11	1.26	17.28	1	50	Mode 1	90
12	1.27	17.28	1	50	Mode 2	90
13	1.28	17.856	1	50	Mode 1	90
14	1.29	17.856	1	50	Mode 2	90
15	1.30	18.432	1	50	Mode 1	90
16	1.31	18.432	1	50	Mode 2	90
17	1.32	19.584	1	50	Mode 1	90
18	1.33	19.584	1	50	Mode 2	90

**Table 4:** Group 3 experiments (12 experiments).

No.	Low Rate Experiment	Motor Position (mm)	Heating Rate (°C/min)	Maximum Temperature Difference (°C)	Experimental Mode	Duration (min)
1	1	19.584	0.6	60	Mode 3	90
2	2	19.584	0.6	60	Mode 4	90
3	3	24.192	0.6	60	Mode 3	90
4	4	24.192	0.6	60	Mode 4	90
5	5	23.04	0.6	60	Mode 3	90
6	6	23.04	0.6	60	Mode 4	90
7	7	21.888	0.6	60	Mode 3	90
8	8	21.888	0.6	60	Mode 4	90
9	9	20.736	0.6	60	Mode 3	90
10	10	20.736	0.6	60	Mode 4	90
11	11	13.824	0.6	60	Mode 3	90
12	12	19.584	0.6	60	Mode 4	90

**Table 5:** Group 4 experiments (8 experiments).

No.	Low Rate Experiment	Motor Position (mm)	Heating Rate (°C/min)	Maximum Temperature Difference (°C)	Experimental Mode	Duration (min)
1	1	24.192	0.6	60	Mode 3	90
2	2	24.192	0.6	60	Mode 4	90
3	3	27.072	0.6	60	Mode 3	90
4	4	27.072	0.6	60	Mode 4	90
5	5	25.344	0.6	60	Mode 3	90
6	6	25.344	0.6	60	Mode 4	90
7	7	24.192	0.6	60	Mode 3	90
8	8	24.192	0.6	60	Mode 4	90

## Data Availability

The data is unavailable due to privacy.

## Appendix A

**Table A1:** Summary of Material/Equipment.

Name of Material/Equipment	Company	Catalog Number	Comments/Description
Silicon oil, 2cSt	Shin-Etsu	KF-96	
Infrared camera	FLIR	Tau2	
Thermocouples, K-type	North University of China	ZBDX-HTTK	The diameters of the bare wires are 0.08 mm and 0.127 mm.
Camera	WATTEC	WAT-30HD	
Hydraulic cylinder	FESTO	ADVU-40-25-P-A	
Motor controller	PI	C-863	
Motor	PI	M-227	
Solenoid valve	FESTO	MFH-2-M5	
Pipe, 4 mm	FESTO	PUN-4X0,75-GE	
Heating film	HongYu	125 Q/W335.1A	
Semiconductor Cooler	Zhongke	9502/065/021M	
LED	693 Institute	10257MW7C	
